# Gut microbiota–cancer crosstalk: emerging molecular targets for personalized cancer treatment- a comprehensive review

**DOI:** 10.3389/fmolb.2026.1924197

**Published:** 2026-09-30

**Authors:** Pooja Negi, Arpita Upreti, Preeti Budhlakoti, Amit Kumar Sen, Mitali Danu, Sumit Tewari, Nirmal Joshi, Deepak Chandra Joshi

**Affiliations:** 1 College of Pharmacy, Graphic Era Hill University, Bhimtal Campus, Bhimtal, Uttarakhand, India; 2 Centre for Promotion of Research, Graphic Era (Deemed to be) University, Dehradun, Uttarakhand, India; 3 Faculty of Pharmaceutical Science, Amrapali University, Haldwani, India; 4 Vasudev College of Pharmacy, Haldwani, India; 5 Department of Pharmaceutical Chemistry, Samrat Prithviraj Chauhan College of Pharmacy, Kashipur, India; 6 Department of Pharmacology, School of Pharmaceutical Education and Research, Jamia Hamdard, New Delhi, India

**Keywords:** artificial intelligence, biomarkers, cancer, gut microbiota, microbial metabolites, personalized medicine, precision oncology, tumor microenvironment

## Abstract

Cancer remains a major global health challenge, and growing evidence indicates that the gut microbiota plays an important role in cancer initiation, progression, metastasis, and response to treatment through complex interactions with host signaling, metabolism, and immunity. This comprehensive narrative review discusses the molecular mechanisms underlying gut microbiota–cancer crosstalk, with particular emphasis on dysbiosis, microbial metabolites, immune modulation, and microbiota-regulated oncogenic pathways, including TLR/NF-κB, Wnt/β-catenin, JAK/STAT, PI3K/Akt/mTOR, and Hippo-YAP signaling. The review also examines emerging molecular targets, including microbial extracellular vesicles, bacterial small RNAs, ferroptosis-related mechanisms, and microbiota–mitochondria interactions. In addition, current microbiota-targeted therapeutic strategies, such as fecal microbiota transplantation, engineered bacterial therapeutics, CRISPR-based microbiome engineering, and microbiome-informed approaches to precision oncology are discussed. Relevant peer-reviewed literature published primarily within the recent 5-year period was considered from major scientific databases and publisher platforms, including PubMed, Scopus, Web of Science, Springer, Elsevier, and MDPI. Overall, the available evidence suggests that gut microbiota represents an important component of the tumor ecosystem and may provide promising biomarkers and therapeutic targets. Integration of microbiome research with multi-omics and artificial intelligence may further support the development of personalized cancer treatment.

## Graphical Abstract

**Figure:**
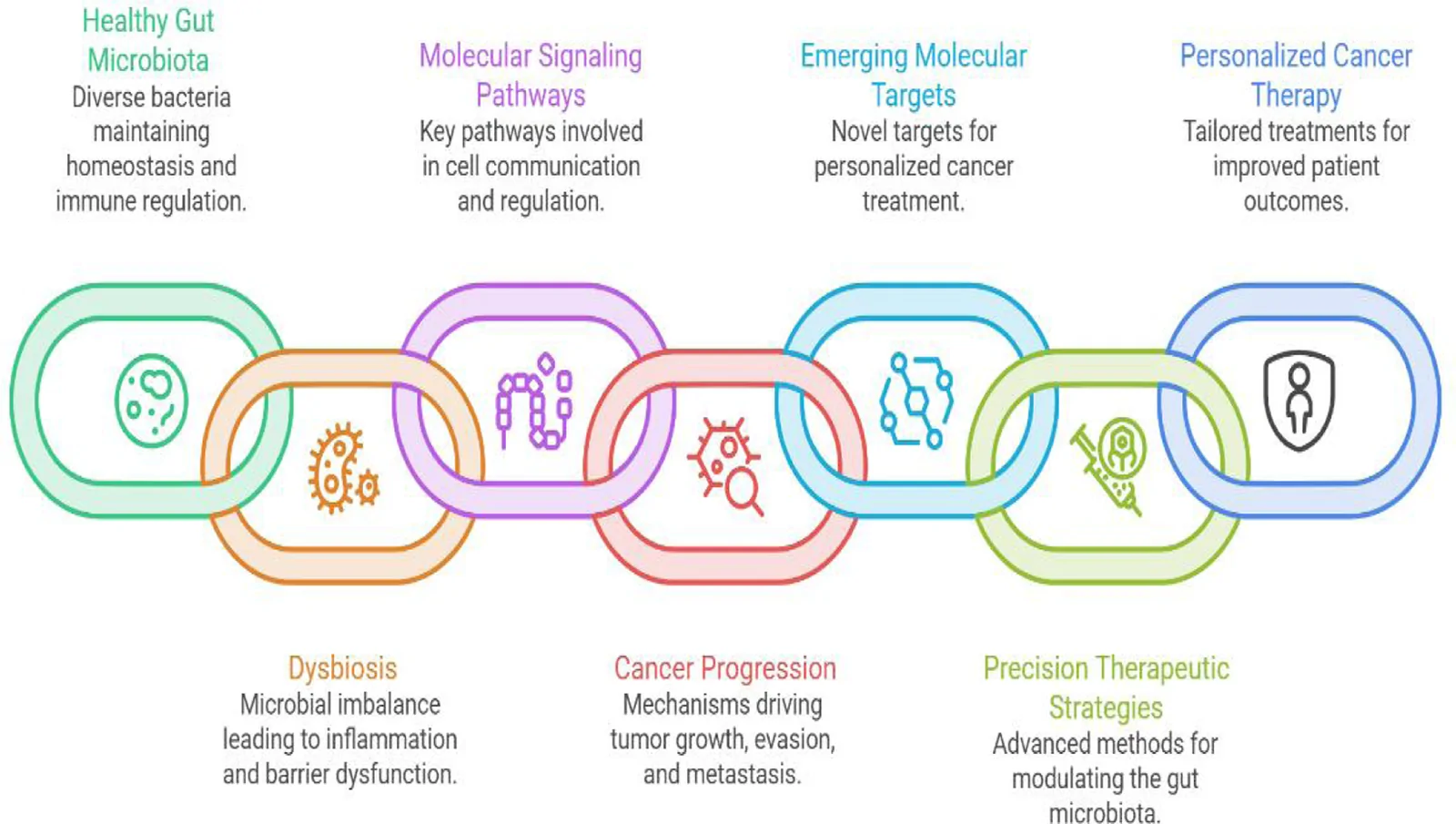

## 1 Introduction

Cancer is a growing global health concern and is a leading global cause of death. Forecasts suggest that cancer incidence will increase as populations grow older, and due to newly emerging lifestyles, environmental changes, and genetic factors (Wu Z. et al., 2024). Resistance to cancer treatment and recurrence of the disease post-treatment remains a significant clinical concern, even with the ongoing development of surgical and non-surgical treatment modalities such as chemotherapy, radiotherapy, targeted treatments, and immunotherapy (Zafar et al., 2025). The human gastrointestinal tract contains a diverse community of microorganisms, called the gut microbiota, that number in the trillions (Rinninella et al., 2019). This community consists of various forms of life such as bacteria, archaea, fungi, and viruses, each able to maintain a beneficial relationship with the host. The gut microbiota plays a vital role in digestion and metabolism, as well as in the maturation of the immune system and the maintenance of the gut barrier, where it protects the host from pathogenic infections (Leonard and Toro, 2023). The development of new sequencing technologies and the multi-omic sciences revealed that dysbiosis, or an imbalanced gut microbiota, is linked to a number of diseases including cancer (Hemmati et al., 2024). The inflammatory response and dysregulation of the immune system and metabolism can contribute to microenvironment changes where tumors can form, whereas microbial populations are able to support immune activation which can produce metabolites that are protective and have anti-tumor functions (Chen et al., 2026). The term “gut microbiota–cancer crosstalk” refers to the two-way line of communication that exists between microbes and tumor cells in which signaling molecules, metabolites, immune mediators, and other cellular pathways of the host are involved (Hu et al., 2025). There is a growing body of literature to support that gut microbiota can influence not only gastrointestinal cancers such as colorectal cancer, but also diverse malignancies including lung, liver, pancreatic, and melanoma, as well as breast cancer (Pandey et al., 2023).

Therapeutic effectiveness is increasingly determined by gut microbiota. Numerous studies have indicated that microbial composition alters patient responses toward immune checkpoint inhibitors, chemotherapy, and targeted therapies. Thus, changing the gut microbiome is a treatment option being researched to improve patient outcomes (Pandey et al., 2023). Growing knowledge of the microbiota and its molecular mechanisms is unlocking additional therapeutic options. These include microbial metabolites, extracellular vesicles, bacterial non-coding RNAs, and microbiome-regulated signaling networks. The foundation is being set for newer, personalized forms of cancer therapy with microbiota-informed treatment and therapeutic approaches (Albano and Taverna, 2025).

For the most part, the gut microbiota-cancer relation is a black box. This review aims to partially elucidate this black box by addressing the available information pertaining the molecular engaging points of gut microbiota with cancer, extending the microbiome-derived therapeutic targets, and analyzing microbiota-targeted interventions in existence. It will include the role of artificial intelligence in microbiome-guided precision oncology (Kouidhi et al., 2023). Figure 1 shows a conceptual framework of the mechanisms that associate tumor development with gut microbial homeostasis, dysbiosis, and microbiota-targeted therapies.

**FIGURE 1:**
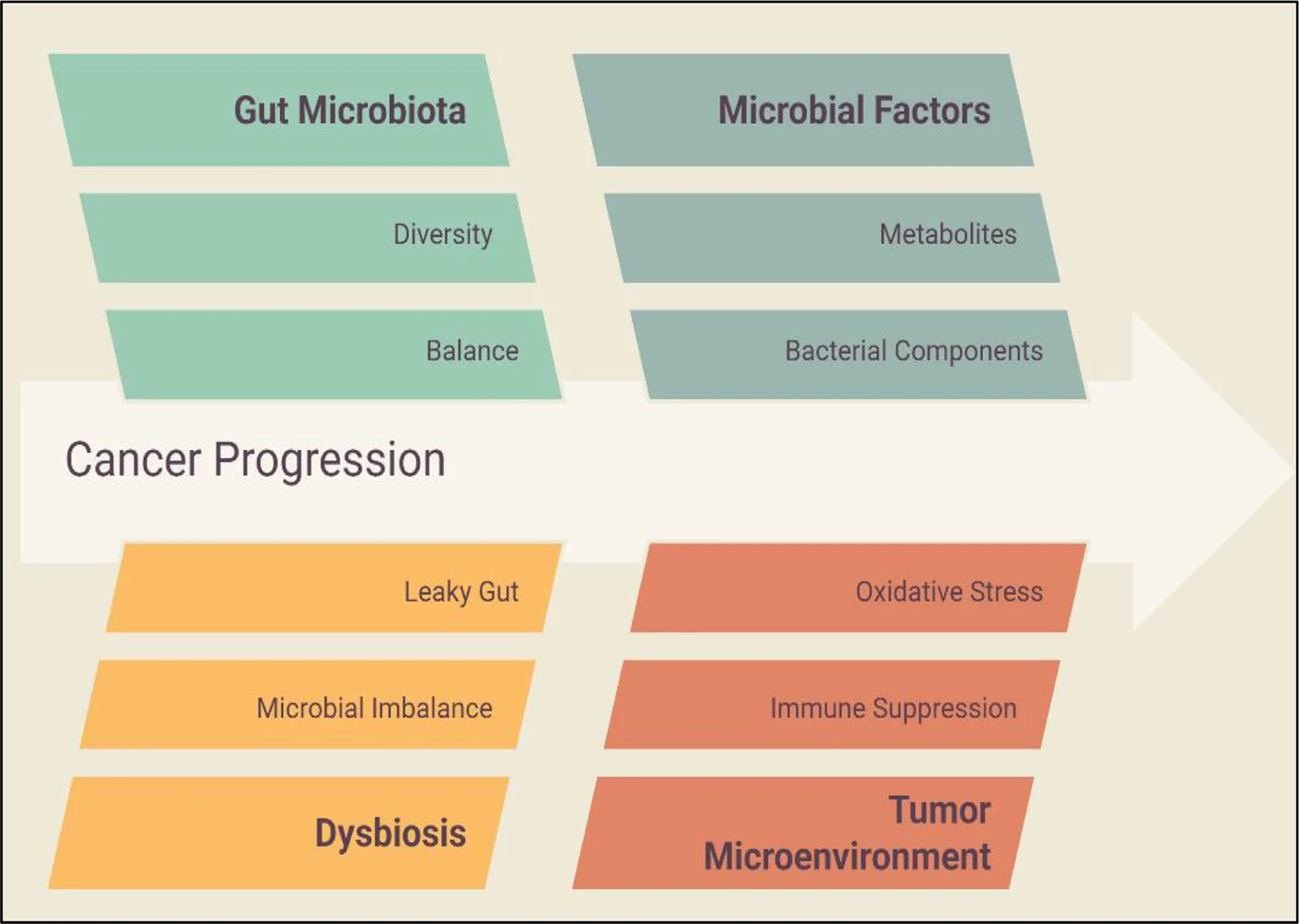
overview of gut microbiota–cancer crosstalk and personalized therapeutic targets.

## 2 Mechanisms linking gut microbiota and cancer development

Because the gut microbiota is able to change host immunity, metabolism, inflammation, and cellular signaling pathways, it is a very important factor in regulating the development of cancer. Intestinal microbiota, under normal conditions, preserves the epithelium and immune system (Li Z. et al., 2024). Dysbiosis, or the disruption of normal microbiota, will result in an imbalance and initiate a chain of processes that cause the formation of a tumor. Many studies support that the active state, as well as the species, consortia, and metabolic activity of different microbiota, is a determining factor in the degree of promotion and inhibition of cancer (Shen et al., 2025). Chronic inflammation is one of the key mechanisms for the promotion of cancer by the gut microbiota. Continued infection by some pathogenic microorganisms will cause release of pro-inflammatory cytokines such as tumor necrosis factor alpha (TNF-α) and interleukins 6 and 17 (IL-6 and IL-17) (Nishida and Andoh, 2025). These inflammatory cytokines help stimulate and activate some of the oncogenic signaling pathways such as NF-κB and the STAT and MAPK pathways and help create microenvironments that are supportive for tumor growth. Chronic inflammation helps promote the constant increase of cells, helps inhibit apoptosis, and helps promote angiogenesis, which, in total, is a greater risk factor for cancer (Zhang et al., 2021). The other big factor is the direct damaging of cells caused by the toxins and metabolites of pathogenic bacteria. Some of the pathogenic bacteria will cause the release of cancer-causing agents which influence the damaging of cells and create an unstable genetic environment. Pks-positive strains of *E. coli* this help causes formation of double strand breaks in DNA and cause genetic mutations. The other pathogenic bacteria, *Bacteroides fragilis*, helps promote the damaging of DNA by causing reactive oxidative stress. These worsen the formation and solvability of cancer-causing agents (Eyvazi et al., 2020; Markelova et al., 2022). Carcinogenesis can be influenced through modulation of epithelial barrier function by gut microbiota. Intestinal dysbiosis causes disruption of tight functions, increasing permeability of the intestinal epithelium. This is known clinically as “leaky gut.” As a result, bacterial components enter the systemic circulation. Lipopolysaccharides, peptidoglycans, and flagellin are components of the leaky gut that cause activation of pattern recognition molecules such as Toll-like receptors and NOD-like receptors, leading to chronically sustained inflammation and the induction of signaling pathways that promote tumorigenesis (Ivleva and Grivennikov, 2022).

Another example of the association between gut microbiota and cancer is through modularity of altered metabolism. Microbiota influences the metabolism of bioactive compounds, which affects tumorigenesis. Short Chain Fatty Acids (SCFAs), which include butyrate, offer metabolic benefits, exerting anti-inflammatory, and anti-proliferative actions through the inhibition of HDAC (Histone Deacetylase) and immune regulation. Alternately, a dysbiotic microbiota can produce secondary bile acids, polysaccharides, and hydrogen sulfide, which promote the production of reactive oxygen species and cellular proliferation, causing DNA damage (Al-Qadami et al., 2022).

The gut microbiota also influences immunity. Alterations of specific gut microbiota influence the recruitment and activation of immune cells and also influence tumorigenesis. Dysbiosis can often promote tumorigenesis by increasing the population of regulatory T-cells, while causing the immune cells to become less cytotoxic. This greatly diminishes the immune response to a tumor (Liu et al., 2024).

Collectively, these findings show that gut microbiota help develop cancer by causing chronic inflammation, increased cellular toxicity, epithelial barrier impairment, metabolic disturbance, and immune dysfunction. These also show that discovering the mechanisms that these gut microbiotas use to develop cancer will lead to the development of new cancer microbiome-based biomarkers and therapeutic targets (Kouidhi et al., 2023). The species of tumors associated with the most prominent cancer-causing microbes and their mechanisms are described in Table 1 **.**

**TABLE 1:** Major cancer-promoting microbial species and their molecular mechanisms.

Microbial species	Associated cancer type	Key virulence factor/Metabolite	Molecular mechanism	Major signaling pathway affected	Ref.
*Fusobacterium nucleatum*	Colorectal cancer	FadA adhesin, Fap2 protein	Promotes tumor cell proliferation, immune evasion, and inflammation	Wnt/β-catenin, NF-κB	(Wu et al., 2019)
Enterotoxigenic *Bacteroides fragilis* (ETBF)	Colorectal cancer	*B. fragilis* toxin (BFT)	Disrupts epithelial barrier, induces IL-17-mediated inflammation	STAT3, NF-κB	(Jasemi et al., 2025)
pks + *Escherichia coli*	Colorectal cancer	Colibactin	DNA double-strand breaks and genomic instability	DNA damage response pathways	(Sadeghi et al., 2024)
*Helicobacter pylori*	Gastric cancer	CagA, VacA toxins	Chronic inflammation, epithelial transformation, apoptosis dysregulation	NF-κB, MAPK, PI3K/Akt	(Duan Y. et al., 2025)
*Peptostreptococcus anaerobius*	Colorectal cancer	Surface adhesion proteins	Enhances ROS production and cholesterol biosynthesis	PI3K/Akt, NF-κB	(Shen et al., 2024)
*Salmonella enterica*	Gallbladder and colon cancer	Endotoxins, effector proteins	Persistent inflammation and DNA damage	STAT3, MAPK	(Mikołajczyk et al., 2024)
*Enterococcus faecalis*	Colorectal cancer	Extracellular superoxide	Oxidative DNA damage and chromosomal instability	ROS-mediated signaling	(Khalyfa et al., 2021)
*Campylobacter jejuni*	Colorectal cancer	Cytolethal distending toxin (CDT)	DNA damage and cell cycle arrest	ATM/ATR signaling	(He et al., 2024)
*Streptococcus gallolyticus*	Colorectal cancer	Adhesion molecules	Promotes proliferation and inflammatory responses	β-catenin pathway	(Kumar et al., 2017)
*Porphyromonas gingivalis*	Oral and pancreatic cancer	Gingipains	Immune suppression and chronic inflammation	NF-κB, JAK/STAT	(Muñoz-Medel et al., 2024)

## 3 Molecular signaling pathways mediating gut microbiota–cancer crosstalk

The gut microbiota communicates with cancer host cellular signaling networks and affects cancer development. This is done via relatively advanced systems of communication. Microbial systems, toxins, and metabolites can engage in the activation of suppressing oncogenic pathways and these further impact cell proliferation and apoptosis, immune surveillance, angiogenesis, and metabolic reprogramming (Sun et al., 2023). There is a lot of evidence showing that the activation of specific cellular pathways resulting from dysbiosis is a major mechanism in connecting the gut microbiome with cancer. Certain pathways, such as pattern recognition receptor signaling, NF-κB, Wnt/β-catenin, JAK/STAT, PI3K/Akt/mTOR, and Hippo-YAP pathways, are key pathways mediating the communication between the microbiome and cancer. These will be further explored in the sections that follow (Kang et al., 2025).

### 3.1 Pattern recognition receptor signaling

Pattern recognition receptors are responsible for the first line of defense in the detection of microbial invasion. This is accomplished by the identification of Molecular Patterns of Microbes. In terms of their study in cancer, the most relevant PRRs are the Toll-like receptors (TLRs) and the NOD-like receptors (NLRs). These receptors are involved in sustaining homeostasis of the intestines, and in the case of TLRs, can contribute to tumor development in the case of dysbiosis (Chen R. et al., 2025). TLRs are found in Epithelial cells, Macrophages, Dendritic cells, and several other immune cells. They have the capability of recognizing bacterial lipopolysaccharides (LPS), flagellin, peptidoglycans, and microbial nucleic acids. The resulting dysbiosis from these factors contributes to the increase in bacterial products which result in the inductive activation of TLR2, TLR4, and TLR9, which induces a chronic inflammatory response. In this case, TLRs engage helper adaptor proteins of the Myeloid differentiation primary response protein 88 (MyD88) type, and these proteins stimulate NF-κB and MAPK pathways (Ciaston et al., 2022). Prolonged activation of TLRs results in the production of inflammatory cytokines that contribute to cell proliferation, angiogenesis, and tumor progression. NOD-like receptors are intracellular detectors of some bacterial cell wall components. NOD1 and NOD2 are implicated in the control of inflammation and epithelial barrier function. Controlled NLR signaling may be important for host defense (Kircheis and Planz, 2023). However, dysregulated activation may cause inflammasome assembly and overproduction of pro-inflammatory cytokines like interleukin1β (IL-1β) and IL-18. The sustained activation of these pathways produces a microenvironment that is pro-tumorigenic. It is likely that NLR and PRR mediated pathways are the most important mechanisms by which the gut microbiota affects cancer (Lara-Reyna et al., 2022).

### 3.2 NF-κB signaling axis

NF-κB is one of the central pathways linking microbial dysbiosis and cancer. NF-κB is a master regulator for inflammation, control of the immune response, cell survival, and promotion of cancer. LPS, flagellin, and bacterial toxins activate NF-κB signaling via TLRs and NLRs. Following activation, IκB proteins are phosphorylated, and subsequently degrade. This allows NF-κB transcription factors access to the nucleus. NF-κB is further activated by the transcription of multiple inflammatory associated genes such as TNF-α, IL-6, IL-1β, COX-2, and iNOS (Mao et al., 2025). The continued activation of NF-κB promotes cellular dysregulation of inflammation and proliferation, as well as the promotion of angiogenesis. Several microorganisms that are associated with cancer such as *Fusobacterium nucleatum*, *Helicobacter pylori*, and Enterotoxigenic *Bacteroides fragilis* activate the NF-κB signaling cascade. Furthermore, NF-κB is associated with apoptosis resistance and immune evasion through its control over inflammatory responses and the expression of negative immune checkpoint proteins. NF-κB is a key pathway linking microbial dysbiosis and tumor inflammatory responses that aid cancer progression (Kearns, 2025).

### 3.3 WNT/β-catenin pathway

Cell growth, development, and tissue repair are all influenced by the Wnt/β-catenin signaling pathway. This pathway can be inappropriately activated by several cancers, including some found in the gastrointestinal tract and the liver and pancreas. Recent research suggests that the gut microbiome can affect Wnt signaling. Certain bacteria can be pathogenic, producing surface-binding proteins and toxins which can interact with and stimulate β-catenin in epithelial cells (Zhao et al., 2022). For example, *Fusobacterium nucleatum* has the FadA adhesin which binds to E-cadherin in epithelial cells and stimulates the β-catenin pathway. Once β-catenin is activated, it can go to the nucleus and stimulate the expression of cancer-related genes like c-Myc and cyclin D1. Inflammation from the microbiome can further stimulate Wnt Signaling by causing the increased production of certain cytokines. Because Wnt signaling is central to colorectal cancer, it is a prime pathway to focus on for antibiotic treatment in cancers related to the microbiome (Wu et al., 2022).

### 3.4 STAT3 and JAK–STAT signaling

The JAK-STAT pathway is involved in the cellular response induced by cytokines and growth factors. Of the STAT proteins, STAT3 is the most crucial in the development and progression of cancer. Microbial dysbiosis of the gut can result in the production of inflammatory cytokines like IL-6, IL-11, and IL-22 (Uivarosi et al., 2026). These activate JAK and result in the phosphorylation of STAT3. Once it is active, STAT3 can enter the nucleus where it controls a variety of genes including ones that can increase proliferation and allow for the progression and spreading of cancer. Enterotoxigenic *Bacteroides fragilis* may lead to the production of IL-17 and IL-6, which causes persistent activation of STAT3 in colorectal cancer. The chronic activation of the JAK–STAT pathway stimulates cancer growth by opposing apoptosis and favoring the survival of cancer stem cells. In addition, STAT3 facilitates the formation of an immunosuppressive tumor microenvironment by manipulating regulatory T cells and myeloid-derived suppressor cells. Thus, at the forefront of innovative precision oncology is the targeting of the microbiome-induced STAT3 signaling (Gargalionis et al., 2021).

### 3.5 PI3K/Akt/mTOR pathway

The phosphoinositide 3-kinase (PI3K)/Akt/mammalian target of rapamycin (mTOR) pathway controls metabolism and growth and is a pathway critical to the proliferation and survival of cells. The majority of malignancies are associated with disturbances in this cascade. Microorganisms and some of their metabolites may activate PI3K/Akt signaling in a receptor-mediated way (Sheng et al., 2026). The CagA protein of *Helicobacter pylori* is one of the microbial proteins which promotes gastric epithelial cellular transformation and apoptosis evasion via the activation of the PI3K/Akt signaling pathway. In a similar manner, *Peptostreptococcus anaerobius* activates PI3K/Akt signaling and facilitates the production of reactive oxygen species and cholesterol. Because mTOR activation favors tumor growth and metabolic alterations and enhances the angiogenic response, and since PI3K/Akt signaling promotes and enhances resistance to targeted therapy and chemotherapy, microbiota-mediated alteration of this pathway explores significant cancer-related concerns such as therapy resistance and tumor progression (Chen et al., 2024).

### 3.6 Hippo–YAP signaling

Regulating the balance between tissue homeostasis and cell proliferation is the conserved Hippo signaling pathway. Aberrations within Hippo signaling are primarily with the activation of oncogenic transcription factors: Yes-associated protein (YAP) and transcriptional coactivator with PDZ-binding motif (TAZ). Emerging evidence suggests gut microbiota plays a role in Hippo-YAP pathway regulation. Microbial metabolites, bacterial-derived components, and inflammatory cytokines participate in the modulation of YAP activity via cellular stress and the integrity of the epithelial barrier. Enhanced YAP activity may lead to stemness and aggressive epithelial–mesenchymal transition and metastatic behavior (Zhong et al., 2024).

In addition, microbial dysbiosis-associated inflammation is capable of augmenting YAP activation in colon and liver cancers. Hippo-YAP, Wnt/β-catenin, and PI3K/Akt pathways have synergistic interactions that promote cancer. The Hippo-YAP pathway’s association with microbiota-related cancer development makes it a valid target for future microbiome-related therapies. These pathways describe how gut microbiota impact cancer. The ability to address microbial and host signaling interactions with YAP and the tumor microenvironment may provide novel targets and ways to treat cancer that is personalized to the individual (Zhang L. et al., 2025). Figure 2 illustrates the oncogenic signaling pathways regulated by microbiota.

**FIGURE 2:**
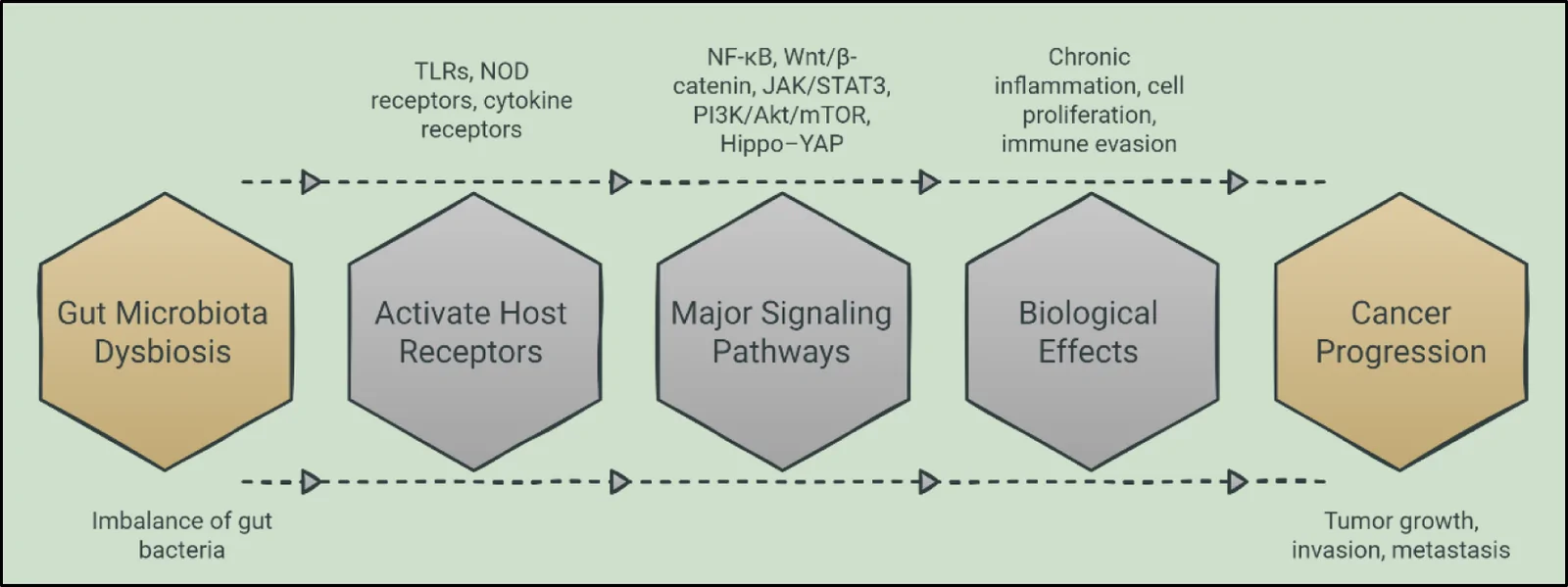
Microbiota-regulated oncogenic signaling pathways.

## 4 Microbial metabolites as emerging molecular targets

Microbial metabolites are important in host–microbiota interactions as well as cancer progression and treatment response. These metabolites are bioactive molecules formed during the microbiota’s metabolism of dietary components, and other substrates and compounds. It is becoming increasingly evident that microbial metabolites act as signaling molecules that can regulate inflammation and immune responses, as well as cellular metabolism and epigenetic changes. Such findings have rendered microbial metabolites of great interest to precision oncology (Mafe and Büsselberg, 2025). Short-chain fatty acids (SCFAs) are among the most notable microbial metabolites. SCFAs are butyrate, acetate, and propionate, and are the products of dietary fiber fermentation by bacteria. Butyrate, in particular, becomes a potent inhibitor of histone deacetylases, and, thus, has the ability to regulate cancer metabolism and promote the differentiation of cancer cells. SCFAs also help to maintain the integrity of the intestinal epithelial barrier and promote the immune system by increasing regulatory T cells and anti-inflammatory cytokines. There is a noted correlation between the reduction of bacteria that produce SCFAs and the occurrence of colorectal cancer and other malignancies (Sankarganesh et al., 2025; Xiong et al., 2022).

Unfortunately, other microbial metabolites can facilitate the development of cancer. Such metabolites are secondary bile acids particularly deoxycholic acid (DCA) and lithocholic acid (LCA). Increased concentration of these bile acid metabolites are associated with colorectal, liver, and other gastrointestinal cancers. Secondary bile acids promote a cancer microenvironment by increasing DNA damage, inflammation, and cellular oxidative stress, as well as instability of the genome. They activate the NF-кB, Wnt/β-catenin, and PI3K/Akt pathways, which induce cancer growth and metastasis (Yang and Qian, 2022). Tryptophan-derived metabolites are important for communication between the host and the microbiome. Gut bacteria convert tryptophan to indole derivatives and activate the aryl hydrocarbon receptor (AhR). AhR is a regulator of immune response and epithelial cell balance. Activated AhR signaling has the potential to control inflammation and protect the mucosal layer. This signaling pathway, when disrupted, can cause immune deficiency and some types of cancer. Manipulating microbial tryptophan metabolism has the potential to improve cancer immunotherapy (Ma et al., 2020).

Another set of metabolites produced by bacteria in the gut are Polyamines, like Spermidine and Spermine. Polyamines are vital for cell growth, but their excess can lead to cancers. Other malignancies in the gut have shown increased levels of polyamines which have the potential to become novel cancer diagnostics and cancer therapies (Li et al., 2025b).

Detrimental effects of metabolites on the immune cancer microenvironment and the efficacy of cancer therapy have been proven. Some metabolites lead to positive responses to immune checkpoint therapy, chemotherapy, and targeted therapies. Because of this, cancer therapy focuses on the metabolites of the microbiome. Microbial metabolites act as a bridge between gut bacteria and the host. While the anti-inflammatory and immunomodulatory effects of several microbial metabolites, particularly short-chain fatty acids, are supported by substantial experimental evidence, direct clinical validation of many proposed metabolite-based therapeutic strategies remains limited. Their potential for regulating cancer and immune metabolism means they can be molecular targets for developing cancer therapies (Li S. et al., 2024). Microbial metabolites that are relevant for cancer treatment are shown in Table 2.

**TABLE 2:** Microbial metabolites and their therapeutic relevance in cancer.

Microbial metabolite	Major microbial source	Molecular target/Pathway	Effect on cancer biology	Therapeutic relevance	Ref.
Butyrate	*Faecalibacterium prausnitzii*, *Roseburia* spp.	HDAC inhibition, GPR43 signaling	Induces apoptosis, inhibits proliferation, reduces inflammation	Potential anti-cancer metabolite and biomarker	(Liu Z. et al., 2025)
Propionate	*Bacteroides* spp., *Veillonella* spp.	GPCR signaling, immune modulation	Anti-inflammatory and immunoregulatory effects	Supports immune homeostasis and tumor suppression	(Dougherty and Jobin, 2023)
Acetate	*Bifidobacterium* spp., *Akkermansia muciniphila*	Cellular metabolism and energy regulation	Modulates immune cell function and epithelial integrity	Potential adjunct in microbiome-based therapies	(Rodrigues et al., 2022)
Deoxycholic Acid (DCA)	*Clostridium* spp.	NF-κB, Wnt/β-catenin pathways	DNA damage, oxidative stress, chronic inflammation	Potential target for prevention of gastrointestinal cancers	(Wu H. et al., 2024)
Lithocholic Acid (LCA)	*Clostridium* spp., *Eubacterium* spp.	FXR and TGR5 signaling	Promotes genomic instability at high concentrations	Therapeutic modulation under investigation	(Chiang and Ferrell, 2020)
Indole-3-Propionic Acid (IPA)	*Clostridium sporogenes*	Aryl Hydrocarbon Receptor (AhR)	Maintains epithelial barrier and immune regulation	Potential immunomodulatory biomarker	(Ren et al., 2025)
Indole-3-Aldehyde (IAld)	*Lactobacillus* spp.	AhR signaling	Suppresses inflammation and promotes mucosal homeostasis	Candidate metabolite for immunotherapy enhancement	(Wang et al., 2023)
Spermidine	*Escherichia coli*, *Bacteroides* spp.	Autophagy and cellular growth pathways	Regulates proliferation and immune responses	Potential therapeutic and prognostic marker	(Truzzi et al., 2023)
Spermine	Gut anaerobic bacteria	DNA stabilization and cell cycle regulation	Excess levels promote tumor progression	Target for metabolic intervention	(Zhang S. et al., 2025)
Hydrogen Sulfide (H_2_S)	*Fusobacterium* spp., sulfate-reducing bacteria	ROS generation and mitochondrial signaling	DNA damage and epithelial dysfunction	Potential target in colorectal cancer	(Lin et al., 2023)
Trimethylamine-N-oxide (TMAO)	Produced indirectly by microbial metabolism	Inflammatory signaling pathways	Associated with systemic inflammation and tumor progression	Emerging biomarker under investigation	(Caradonna et al., 2025)

## 5 Gut microbiota and tumor immune microenvironment

Tumor immune microenvironments (TIMEs) contain an array of constituents including tumor cells, immune cells, and stroma, as well as cytokines and components of the extracellular matrix, making them complex and dynamic systems. Recently, gut microbiota have been shown to affect tumor microenvironments by influencing immune responses that impact tumor growth and the efficacy of tumor-targeting therapies. Gut microbiota can influence the differentiation and functional responses of immune cells of the local and systemic compartments through the production of microbial metabolites as well as structural components and signaling molecules (Henke et al., 2019). A diverse and balanced microbiota helps to facilitate antitumor immune responses by supporting the maturation of professional antigen-presenting cells (APCs) and the generation of cytotoxic immune responses. In the context of gut microbiota, commensal bacteria can activate dendritic cells, making them capable of presenting foreign antigens to T lymphocytes, which in turn results in the generation of CD8^+^ cytotoxic T lymphocytes. Some of these beneficial bacteria can be linked with the enhanced activity of natural killer (NK) cells, which broadens the immune response to newly forming tumors (Xie and Liu, 2024).

On the other side of the equation, the imbalance of gut microbiota can be supportive of immune microenvironments that foster tumor growth. A shift in gut microbiota from a symbiotic to a dysbiosis state can lead accelerated immune tolerance and a suppressed state of anti-tumor immunity. In this state of immune dysregulation, the composition of the immune response is dominated by regulatory T cells (Tregs) and M2 macrophages, as well as myeloid derived suppressor cells (MDSC). In this dysregulation of the immune microenvironment, tumor progression, local invasion and metastasis can also be supported (Yurtseven et al., 2025).

The gut microbiome also shapes networks of cytokines that control communications between immune cells within tumors. Microbial signals can influence cytokines such as IL-10, IL-12, IFN-γ, and TGF-β, resulting in diverse immune activation versus suppression. Alterations to these cytokines can influence how a tumor acts, as well as the advancement of a disease (Farhadi Rad et al., 2024).

Perhaps the most important balance of microbiota and immune system components for the discussion of cancer is on the influence of cancer immunotherapy. There are many studies indicating that the microbiome can change how patients respond to immunotherapy that blocks PD-1, PD-L1, and CTLA-4. Patients who contain the most beneficial microbiota have the most good responses to treatment, the longest progression-free responses to treatment, and the most beneficial responses in the immune system. In contrast, loss of diversity in the microbiota is tied to a negative response to treatment and the emergence of resistance (Yu et al., 2024).

However, the influence of gut microbiota on immune checkpoint inhibitor efficacy is highly heterogeneous and should not be interpreted as uniformly beneficial. Considerable inter-patient variability exists because microbiome composition is affected by diet, geography, ethnicity, age, obesity, antibiotic exposure, proton pump inhibitor use, and prior anticancer treatments. Consequently, microbial taxa associated with favorable immunotherapy responses in one cohort are not always reproducible in independent populations. In addition, several mechanisms may contribute to microbiome-associated resistance to immune checkpoint blockade, including impaired antigen presentation, reduced dendritic-cell maturation, inadequate CD8^+^ T-cell priming, expansion of regulatory T cells and myeloid-derived suppressor cells, altered interferon signaling, and production of immunosuppressive microbial metabolites. Emerging evidence also indicates that microbial translocation and chronic low-grade inflammation may promote T-cell exhaustion and dysfunctional antitumor immunity in some settings. The immunologic consequences of microbiota modulation are further complicated by tumor-type-specific factors. Tumors with high baseline immune infiltration, such as melanoma and some mismatch-repair-deficient cancers, may be more susceptible to microbiome-mediated enhancement of immunotherapy, whereas immunologically “cold” tumors, including many pancreatic and certain colorectal cancers, may exhibit limited responsiveness despite microbiome manipulation. Moreover, excessive immune activation induced by microbiota-directed interventions could theoretically increase the risk of immune-related adverse events, emphasizing the need for careful patient selection and immune monitoring. These observations underscore that microbiome–immunotherapy interactions are context dependent and that personalized approaches will be required to optimize microbiome-guided immuno-oncology (Guo et al., 2024).

There is evidence that the incorporation of microbes can influence the reorganization of the immune system within the microenvironment of the tumor. These changes can influence how immune cells move in and out of the microenvironment, how they secrete cytokines, and how they express checkpoint molecules. This research is beginning to consider how changes in gut microbiota can influence how immunotherapy is applied (Guo et al., 2025; Zhu et al., 2025). Overall, gut microbiomes are a cornerstone for the immune system and tumors. It can influence how communications are formed at the level of the immune system and the tumor. It is now being considered for the creation of personalized immunotherapy in cancer, as well as to develop new biomarkers (Charles and Thomas, 2023). Figure 3 illustrates the Microbiota-Immune-Tumor Triad in Precision Oncology**.**

**FIGURE 3:**
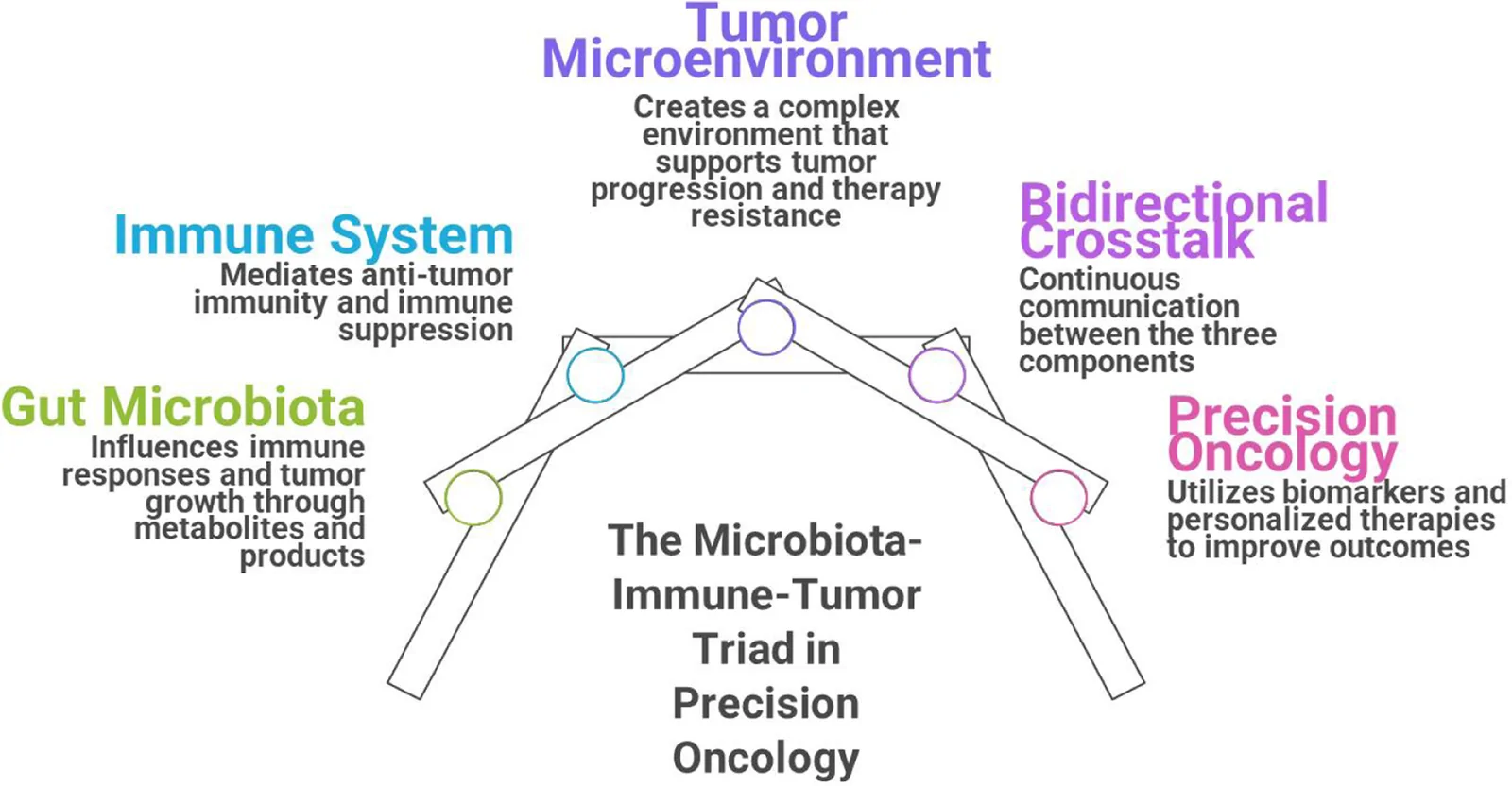
Microbiota-immune-tumor triad in precision oncology.

## 6 Emerging molecular targets for personalized cancer therapy

Advancements in microbiome science have sparked new interest in the realms surrounding the relationship between gut microbiota and cancer. Research suggests that the role of gut bacteria in tumor progression is greater than that of inflammation and metabolism. Different ways of regulating the formation of extracellular vesicles, non-coding RNAs, and ferroptosis, as well as mitochondrial function, suggest influences at a greater therapeutic level. These methods could result in the development of new, more personalized cancer therapies. In contrast to other therapies that attempt to maximize the alteration of the gut microbiome, this strategy is aimed at mediators of host-microbe interactions at a molecular level, thus providing a greater therapeutic window and a high degree of specificity (Zhao et al., 2023).

### 6.1 Microbial-derived extracellular vesicles (MEVs)

MEVs are small extracellular vesicles produced by members of both the Gram-positive and Gram-negative bacterial Phyla. These vesicles can have a diverse cargo that elicits therapeutic effects due to bioactive RNAs. Due to their size, stability, and ability to carry a variety of cargo, MEVs can communicate across barriers. MEVs play a role in cancer communication by impacting the immune system and by communicating directly to epithelial cells and other cells in a cancer environment. MEVs can have tumorigenic effects or can impact tumors in other ways depending on their bacterial sources. Pathogenic bacterial vesicles can cause inflammation and immune signaling and proliferation of cells, whereas beneficial bacterial vesicles can provide support for immune surveillance (Fang et al., 2022).

Another benefit of MEVs is their possible use as diagnostic biomarkers. MEVs contain cargo that reflects the activity of microbes and changes associated with disease in the intestinal biosystem. MEV profiling could allow for the early detection of cancer and the monitoring of treatment. In addition, engineered bacterial vesicles are being studied as a means of targeted drug delivery, transporting therapeutic molecules directly to tumor tissues and avoiding systemic effects of toxicity (Yang, 2024).

### 6.2 Bacterial small RNAs (bsRNAs)

Bacterial small RNAs (bsRNAs) are short, non-coding RNA molecules that can regulate gene expression in bacterial cells. New studies are showing that these molecules can also regulate the functions of host cells, allowing them to communicate across different biological kingdoms. bsRNAs can be transmitted to mammalian cells via extracellular vesicles, and directly from microbe to host. It has been shown that certain bacterial RNAs can regulate genes that are involved in inflammation, apoptosis, and tumor formation (Ponath et al., 2022). These molecules are capable of changing the transcript of epithelial, as well as immune, cells and thus affect the microenvironment of a tumor. Unlike traditional metabolites derived from microbes, bsRNAs offer a specific means to alter cellular response and communicate at the post-transcriptional level. bsRNAs can be used for therapies because they can control disease-modified pathways. New technologies in RNA sequencing and transcriptomics will allow for the determination of RNA signatures of the microbiota in different cancer types. These signatures will help develop prognostic biomarkers that will predict the progression of cancer and responses to treatment. Furthermore, new strategies in RNA to copy the functions of a microbe will help formulate new ideas in precision oncology (Duan H. et al., 2025).

### 6.3 Gut microbiota–CircRNA–miRNA networks

CircRNAs and miRNAs are non-coding RNAs that have significant roles in the regulation of gene expression after transcription. Recent evidence suggests that gut microbiota can affect the expression of circRNAs and miRNAs in the host, establishing sophisticated networks with consequences on cancer. CircRNAs act as sponges by sequestering miRNAs. As a result, circRNAs can control the access of miRNAs to target mRNAs. Changes in circRNA expressions due to alterations in the microbiota can have knock-on effects on the regulatory networks for gene expression involved in cell proliferation, differentiation, angiogenesis, and metastasis. Microbial metabolites and other signaling molecules can affect a number of oncogenic and tumor-suppressive miRNAs (Zhu et al., 2020). The microbiota–circRNA–miRNA pathway has emerged as an important component of host–microbe interaction. These networks, because of their complex nature, are indicative of a specific tissue and disease state, and therefore can be used as cancer biomarkers to guide diagnosis, prognosis, and treatment. Combining microbiome and transcriptome studies can generate targeted therapies for specific alterations in cancer (Dawoud et al., 2024).

### 6.4 Microbiota-regulated ferroptosis pathways

Ferroptosis is a form of cell death that is regulated and is a consequence of lipid peroxidation that is dependent on iron. This death pathway is distinct from apoptosis and necrosis in that ferroptosis is largely controlled by the redox state of the cell and the balance of iron and lipids. Recent studies have shown that gut microbiota may affect the susceptibility of cells to ferroptosis by modulating inflammatory and metabolic pathways (Čepelak et al., 2020). Microbial communities help maintain iron balance in hosts and release metabolites that influence the response to oxidative stress. Dysbiosis may impact the concentration of iron and reactive oxygen species and antioxidants, and thus the ferroptosis signaling of tumor cells. Microbial metabolites can influence the metabolism of glutathione, lipid peroxidation and ferroptosis-related enzymes such as glutathione peroxidase 4 (GPX4) (Ding et al., 2025).

Microbiota and ferroptosis are interconnected in the context of treatment-resistant cancers. The stimulation of ferroptosis is an attractive approach to targeting malignant cells that resist standard therapies. Hence, the influence of microbial components on ferroptosis may provide better understanding for developing new combination therapies to improve the effectiveness of chemotherapy, radiotherapy and immunotherapy. As knowledge continues to grow in this area, the microbiota-regulated ferroptosis is expected to become a focal point of cancer research. Mitochondria are the key regulators of cellular energy, redox balance and apoptosis (Cai et al., 2025). They are evolutionarily derived from bacteria, thus providing an area for host-microbe interactions. The function of mitochondria may be significantly altered by gut microbiota in a number of ways, from microbial metabolites to immune-mediated mechanisms. Microbial products may impact mitochondrial respiration, the synthesis of ATP and the generation of reactive oxygen species. Beneficial microbial metabolites may help sustain the health of mitochondria, whereas signals from dysbiosis may lead to a decline in mitochondrial health and flexibility. Such changes may help sustain the transformation of cells, promote the survival of malignant cells and increase resistance to anticancer therapies (Zachos et al., 2024).

Cancer’s association with mitochondrial dysfunction is now well established. Targeting cancer through microbiota-controlled mitochondria is a possibility. Microbial composition and microbiota metabolite manipulation can help restore mitochondrial equilibrium. This, in turn, may increase the efficacy of treatment. The combined study of microbiome and mitochondrial biomarkers may shed light on the prognosis and treatment response of the disease. These developing targets highlight the intricacies of cancer and gut microbiota interplay. Extracellular vesicles, bacterial small RNAs, thriving bacterial non-coding RNA regulatory networks, ferroptosis, and mitochondrial communication systems are some of the many emerging and unexplored elements of gut and cancer microbiota. Studying these targets may give rise to new microbiome-based cancer therapies (Weber-Stiehl et al., 2022). Novel targets being investigated in clinical or preclinical studies are shown in Table 3.

**TABLE 3:** novel molecular targets under clinical and preclinical investigation.

Emerging target	Primary mechanism	Impact on cancer biology	Potential clinical application	Current status	Ref.
Microbial-Derived Extracellular Vesicles (MEVs)	Transfer of proteins, nucleic acids, metabolites	Immune modulation, signaling regulation	Biomarkers, targeted drug delivery	Preclinical	(Yang et al., 2018)
Bacterial Small RNAs (bsRNAs)	Inter-kingdom gene regulation	Control of inflammation and tumor-associated genes	Diagnostic and prognostic biomarkers	Preclinical	(Ponath et al., 2022)
CircRNA–miRNA Networks	Post-transcriptional gene regulation	Regulation of proliferation, angiogenesis, metastasis	Precision diagnostics and patient stratification	Experimental	(Frazzi et al., 2025)
Microbiota-Regulated Ferroptosis	Modulation of iron metabolism and lipid peroxidation	Ferroptotic cell death and therapy sensitization	Combination cancer therapies	Preclinical	(Wen et al., 2025)
Microbiota–Mitochondrial Crosstalk	Regulation of mitochondrial metabolism and redox balance	Metabolic reprogramming and treatment response	Therapeutic targeting of cancer metabolism	Emerging	(Li et al., 2025a)
Microbiome-Derived Metabolomic Signatures	Metabolic pathway modulation	Prediction of tumor behavior	Precision oncology biomarkers	Clinical evaluation	(Malczewski et al., 2020)
Engineered Microbial Vesicles	Targeted delivery of therapeutic payloads	Enhanced tumor specificity	Drug and gene delivery platforms	Preclinical	(Ming-Kun et al., 2024)
Microbiome-Based Multi-Omics Targets	Integration of genomic, transcriptomic, and metabolomic data	Personalized treatment prediction	Precision medicine platforms	Early clinical investigation	(Duan et al., 2024)

## 7 Gut microbiota as a biomarker for precision oncology

The intrapersonal variability of cancer and the variability in how patients respond to cancer treatment and therapies has triggered the hunt for reliable biomarkers for the field of precision oncology. The gut microbiota has been a focus of the precision oncology community due to the dynamic interactions the gut microbiota establishes with host metabolism, immune response, and the effects of therapies. The cancer research community in the age of precision oncology has at its disposal a host of powerful technologies (next-generation sequencing, metagenomics, etc.) that facilitate the study of unique cancer associated microbial communities (Li Z. et al., 2024). Many patterns of dysbiosis (including decreases and increases in the diversity of microbial communities) are associated with an increased susceptibility to cancer and the later stages of cancer. Certain malignancies have specific patterns of enriched and diminished microbial communities. Especially for early cancer detection and the subsequent risk and prognostic assessment, these patterns have utility. Differing from traditional biomarkers that rely on the removal of cancer-affected tissues, the biomarkers of the microbiome, require the collection of stool samples, facilitating the adoption of a new standard of care (Goswami and Bose, 2024).

Gut microbiota has potential utility beyond being a cancer detection biomarker; it has utility as a predictor of response to therapy. Several published works have demonstrated that the microbial communities of the host that are present before therapy, and particularly before the initiation of various therapies, are critical to determining the outcomes of various cancer therapies. These suboptimal communities of the gut microbiota also have been associated with toxicity of the therapy and cancer recurrence. Therefore, microbiome analysis has the potential utility of determining responders to specific therapies (Eslami et al., 2025). The clinical utility of microbiome-associated biomarkers is augmented with the integration of other -omics datasets such as genomics, transcriptomics, proteomics, and metabolomics. Integration of microbiome data with host genomics, transcriptomics, proteomics, metabolomics, and epigenomics enables a systems-level understanding of tumor biology. Genomic profiling can identify host susceptibility loci and oncogenic mutations that interact with microbial communities, whereas transcriptomic analyses reveal immune activation signatures, inflammatory pathways, and microbiota-responsive gene expression patterns. Proteomic approaches provide information regarding cytokines, immune mediators, and signaling proteins that reflect functional changes within the tumor microenvironment. Metabolomic profiling identifies microbial- and host-derived metabolites, including short-chain fatty acids, bile acids, indole derivatives, and polyamines, that influence cancer progression and therapeutic responsiveness. The combined analysis of these datasets using integrative bioinformatics and machine-learning approaches improves biomarker accuracy, facilitates patient stratification, predicts response to immunotherapy and chemotherapy, and enables dynamic monitoring of treatment-associated microbiome alterations. Such multi-omics frameworks are expected to play a central role in the development of clinically actionable microbiome-guided precision oncology platforms (Cao et al., 2024).

There are still many challenges, despite the promising concepts. These challenges include poor standardization of sample collection, sequencer methods, analytical frameworks, and sample population characteristics. Reconciliation of the above issues is required to enable clinical practice. The gut microbiome has the potential to be an excellent addition to a novel biomarker platform. The gut microbiome is informative and has the potential to be minimally invasive to support the personalization of cancer diagnostics, prognostics, treatment, and the surveillance of cancer. The gut microbiome integrated into precision oncology may offer more advanced cancer care (Filardo et al., 2024).

## 8 Microbiota-targeted therapeutic strategies

There is more evidence for the role of gut microbiota in cancer. This evidence has led to the development of microbiota-targeted interventions. While conventional anticancer therapies focus solely on cancerous cells, these therapies focus on changing the microbial ecosystem or leveraging microbial functions for therapeutic action. Importantly, the strength of evidence varies considerably across microbiota-targeted interventions; fecal microbiota transplantation has entered early clinical evaluation in selected cancer settings, whereas many engineered bacterial and CRISPR-based approaches remain predominantly preclinical. Innovative strategies such as fecal microbiota transplantation (FMT), engineered bacterial therapeutics, and CRISPR-based Microbiome Modifications and Engineering have emerged as a result of the rapid developments in Microbiome Science. These innovative strategies have placed Microbiome Science in the forefront of Precision Oncology, as they allow the modification of Host-Microbe interaction at a greater level than previously achieved (Cheng et al., 2020).

### 8.1 Fecal microbiota transplantation (FMT)

Fecal microbiota transplantation is the transfer of processed fecal matter to the gastrointestinal system of a recipient in order to restore the microbial diversity and ecosystem balance in the gut. While the original application of FMT was to treat recurrent *Clostridioides difficile* infection, FMT is gaining other therapeutic interests in the Oncology field. Preclinical and clinical studies suggest that FMT positively impacts the immune system’s response to cancer (anti-tumor immunity), and helps to improve the efficacy of cancer treatment that employs immune checkpoint blockade. One of the differences in patients who benefit from Immunotherapy is that they are more likely to have an enriched and diverse gut microbiota with a balance of good bacterial communities. There are a few documented cases of patients who did not respond to treatment, and after FMT, they demonstrated a moderate improvement in the efficacy of the therapeutic interventions that they received (Baruch et al., 2021; Liu Y. et al., 2025). Using fecal microbiota transplantation (FMT) could have a positive therapeutic outcome when combined with immunotherapy and could mitigate some adverse gastrointestinal effects from immunotherapy and bolster intestinal barrier function. Concerns about the selection of donors, safety of the recipients, standardization of the procedure, and a sustained positive effect from the transplantation are issues that still need to be addressed. The future may include a microbial consortium that is more diverse (and more controlled) than standard FMT and may be more effective while maintaining FMT’s benefits (Wang X. et al., 2025).

### 8.2 Engineered bacteria for cancer therapies

Synthetic biology has made it possible to genetically modify bacteria so that they can preferentially seek out cancerous tumors and deliver medicine. Traditional systems used for drug delivery cannot fully leverage the unique abilities of bacteria to seek out the hypoxic, nutrient-dense regions within a tumor. Bacteria have a unique advantage of targeting a tumor and remaining localized while providing therapeutic systems that are of reduced exposure to the rest of the body and therefore have fewer side effects. There are also systems that can be designed to enhance presentation and loading of tumor antigens, thereby promoting a robust immune response to the tumor. Designed systems have a great safety profile and will offer more precision to treatment than standard therapies. Although numerous engineered bacterial therapies are in preclinical and early clinical studies, more work to optimize the systems for safety will be required before they are used more broadly in a clinical setting (Chang et al., 2025).

Despite their therapeutic promise, engineered bacterial platforms face several major translational challenges. Efficient delivery to tumor tissues remains a critical limitation because systemically administered bacteria are often rapidly cleared by the reticuloendothelial system, and intratumoral colonization can vary substantially according to tumor vascularization, hypoxia, immune status, and anatomical location. Biosafety is another central concern, particularly in immunocompromised cancer patients, because even attenuated bacterial strains may cause bacteremia, sepsis, systemic inflammation, or unintended tissue colonization (Wang Y. et al., 2025). Genetic stability must also be carefully controlled, as therapeutic constructs may lose functionality during replication or undergo mutation, recombination, or horizontal gene transfer within the host microbiome, potentially altering efficacy or safety. Strategies such as auxotrophic mutations, kill-switch circuits, inducible expression systems, and containment mechanisms are being developed to reduce these risks, but long-term *in vivo* stability data remain limited. Additional barriers include scalable Good Manufacturing Practice (GMP) production, batch-to-batch consistency, storage stability, quality control, and regulatory classification of living microbial therapeutics. Furthermore, clinical translation requires optimization of dosing schedules, routes of administration, patient selection criteria, combination strategies with immunotherapy or chemotherapy, and standardized monitoring for microbial persistence and immune-related adverse events. These unresolved issues indicate that engineered bacterial therapies, although highly promising, should currently be considered emerging investigational approaches rather than established clinical interventions (Gulig et al., 2024).

### 8.3 CRISPR-based microbiome engineering

CRISPR technology creates the ability to edit genes. It has application in precise alterations to collection of microbiome. Development of CRISPR technology allows developers to modify specific desired species of microbiome dysbiosis. Where traditional methods develop all the species of microbiome, CRISPR technology addresses a specific target(s) of microbial species, pathways of microbial genes or virulence of microbials to treat diseases. Engineering of CRISPR technology allows developers to target specific bacteria of the microbiome which are carcinogenic in nature while not altering the overall beneficial members of the Microbiome. CRISPR technology allows developers to target specific microbial genes which contain the signaling of inflammation, antimicrobial resistance, production of toxins and/or carcinogenic metabolic pathways (Ansori et al., 2023). CRISPR technology enables developers to target specific carcinogenic signaling pathways bacteria of the Microbiome. CRISPR technology allows the manipulation of microbes to retain the production of beneficial bacteria to enhance the production of therapeutic metabolites, immune-modulating molecules or anticancer proteins. Development of CRISPR technology along the lines of synthetic biology expands the frontiers of design and development of advanced living therapeutics which are microorganisms tailored and customized to suit individual therapeutic requirements (Amen et al., 2025).

Despite the huge advantages and various functionalities of CRISPR technology, many obstacles remain to be overcome in the CRISPR field. These include the ways to address precise delivery of CRISPR, eco-impacts of CRISPR, and long-term consequences of engineered microbes. However, the rapid advancements in the field of gene technology will continue to pave the way for more precise methods of microbiome interventions. The combination of fecal microbiota transfer (FMT) engineered microbial therapeutics, and CRISPR technology to modify the Microbiome represents groundbreaking, innovative methods to improve and maximize the healing properties of the gut Microbiome. Further tailoring and personalizing these methods to specific patient populations may provide new, innovative cancer therapeutics to improve resistance-based cancer therapies (Tang et al., 2025). As shown in Table 4, current cancer research and novel ways to use microbiome therapeutically are included.

**TABLE 4:** Current clinical trials targeting the gut microbiome in cancer.

Therapeutic strategy	Representative candidate/ Approach	Mechanism of action	Cancer Type(s) investigated	Development status	Ref.
Fecal Microbiota Transplantation (FMT)	Responder-to-patient FMT	Restores microbial diversity and improves immunotherapy responsiveness	Melanoma, colorectal cancer	Clinical trials	(Wekking et al., 2025)
Defined Microbial Consortia	VE800	Administration of selected commensal bacterial strains to enhance CD8^+^ T-cell activity	Solid tumors	Phase I clinical trials	(Tanoue et al., 2019)
Microbiome Therapeutics	SER-155	Modulation of gut microbial composition and immune responses	Hematologic malignancies	Clinical evaluation	(Chen et al., 2026)
Microbiome Ecosystem Therapy	MaaT013	Restoration of microbiome diversity and immune homeostasis	Acute graft-versus-host disease and cancer-associated complications	Clinical trials	(Chen Z. et al., 2025)
Live Biotherapeutic Product	MRx0518	Immune activation through microbial signaling pathways	Solid tumors	Phase I/II clinical studies	(Pant et al., 2020)
Engineered Bacterial Therapy	Modified *Escherichia coli* Nissle 1917	Targeted production of therapeutic molecules within tumors	Colorectal and solid tumors	Preclinical/Early clinical	(Yu et al., 2020)
Tumor-Targeting Bacterial Vectors	Attenuated *Salmonella* strains	Selective tumor colonization and localized therapeutic delivery	Breast, colorectal, pancreatic cancers	Clinical investigation	(Park et al., 2025)
Probiotic-Based Adjuvant Therapy	*Lactobacillus* and *Bifidobacterium* formulations	Enhancement of mucosal immunity and reduction of treatment-associated toxicity	Gastrointestinal cancers	Clinical studies	(Kaur, 2025)
CRISPR-Based Microbiome Engineering	Gene-edited microbial populations	Elimination of pathogenic genes and carcinogenic microbial functions	Multiple cancer models	Preclinical	(Amen et al., 2025)
Engineered Probiotic Platforms	Synthetic biology-derived probiotics	Production of cytokines, metabolites, or anticancer proteins	Solid tumors	Experimental	(Muhammad et al., 2026)
Bacteriophage-Mediated Microbiome Editing	Targeted phage therapy	Selective depletion of cancer-promoting bacterial species	Colorectal cancer	Preclinical	(Kenneth et al., 2025)
Personalized Microbiome Intervention	AI-guided microbiome modulation	Individualized microbial optimization based on patient-specific profiles	Multiple cancers	Emerging precision oncology approach	(Dragomir et al., 2026)

## 9 Artificial intelligence and machine learning in microbiome-guided cancer therapy

The promising evolution of microbiome study has compounded ample intricate biology and data, burdening interpretative and translational aspects. Advanced computational capabilities, such as artificial intelligence (AI) and machine learning (ML), have demonstrated enhanced effectiveness in identifying concealed trends in multidimensional data concerning microbiomes. Aid of microbial, genomic, transcriptomic, metabolomic, and clinical data is paving the way for microbiome-centered precision oncology (Dakal et al., 2024).

ML can be applied to ascertain microbial patterns or profiles that may pose a higher risk for cancer, affect the progression of cancer, or impact treatment and its outcome. In comparison to traditional statistical methods, ML is capable of analyzing more expansive datasets and can explain the complex interrelations of microbial communities, host factors, and tumor characteristics. Predictive models of this nature have considerable effectiveness in the early detection of cancer and the stratification of patients (Pennisi et al., 2025).

AI is being applied to estimate the probability of response to specific classes of chemotherapy and immunotherapy, as well as targeted therapies. Research has established a considerable relationship of microbiome structure and dynamics with the outcome and adverse events of therapy. The inclusion of microbiome-derived bio-signatures into predictive models may improve the precision of treatment and avoidance of adverse events for a specific therapy (Shoji et al., 2022).

Another novel use case is the incorporation of advanced AI systems in the integration of multiple layers of omics data. These systems have the capability to analyze microbial communities, host and perturbed molecular, and immune and metabolic frameworks, thereby establishing comprehensive models of diseases. These analyses promote a more extensive understanding of Host–Microbiota interactions and facilitate the identification of new therapeutic targets (Shen et al., 2026).

AI-centered platforms are likely to create interventions that are geared toward one’s specific microbiome. These include the design of tailored probiotic formulations, microbiome-targeted interventions, and personalized therapies. As the computational world evolves, so does the role of digital microbiome profiles in the realm of clinical coding (Alexandrescu et al., 2025).

There are roadblocks to clinical acceptance despite the promise that AI, machine learning, and microbiome science have as a trifecta. Data standardization, interpretability of modeling, diversity of sampling, and exogenous validation are some of the obstacles that hinder clinical implementation. The combined power of AI, machine learning, and microbiome science is likely to change the future of cancer diagnosis, prognosis, and therapies. These integrated systems of technology will facilitate the design of personalized therapies. They will improve the chances of developing the cancer patient in a better way with newer oncological therapeutics (Khelfaoui et al., 2026).

## 10 Challenges and future perspectives

Although there are now more opportunities to examine the connection between gut microbiota and cancer, several scientific, technical, and clinical issues hamper the introduction of microbiome discoveries to the oncology field. The major challenge is the variation of gut microbiota between individuals. It can be due to the genetics, age, diet, lifestyle, region, medication, and environmental factors of an individual. This variation makes it impossible to find a standard microbial biomarker and therapeutic target to be used on different populations (Min et al., 2026).

Another key issue is the absence of a standard set of protocols and target procedures for sample collection, preparation, and sequencing, including the methodologies for data interpretation. This absence makes the experimental results inconsistent and irreproducible and makes comparisons between studies unfeasible. Furthermore, the majority of current data rests on associative studies, which makes establishing microbial alterations and cancer association impossible. For this purpose, extensive and high-quality research is necessary to study the microbiome and define its targets in cancer (Kapoor and Narayanan, 2023). There are also safety concerns. Microbiota-targeted interventions, such as fecal microbiota transplantation, engineered bacteria, and microbiome editing technologies, are promising although the effects on the physiology of the host and the ecology of the microbiome will be unknown for a long time. The risks, for instance, unintentional alterations to the microbiome, transfer of genes, and immune disturbances, need to be investigated before they can be used routinely in practice (Ramesh et al., 2026).

A major limitation of the current literature is that much of the evidence linking gut microbiota to cancer originates from preclinical studies, including germ-free animal models, antibiotic-depletion experiments, and *in vitro* systems, whereas robust prospective human studies remain comparatively limited. Associations between specific microbial taxa and cancer often vary across cohorts because of differences in diet, geography, ethnicity, medication exposure, sequencing platforms, and bioinformatic pipelines, leading to inconsistent findings and limited reproducibility (Guo et al., 2024). Furthermore, many reported microbial signatures are correlative rather than causal, and it remains difficult to determine whether dysbiosis is a driver of tumorigenesis, a consequence of cancer, or a reflection of treatment-related changes. Similar limitations apply to microbial metabolites and signaling pathways, for which mechanistic evidence is frequently derived from experimental models without confirmation in human tissues or clinical settings. Although microbiome-targeted interventions such as fecal microbiota transplantation, defined microbial consortia, engineered bacteria, and CRISPR-based microbiome editing show promise, most approaches are still in preclinical development or early-phase clinical trials, and long-term safety, durability of response, optimal patient selection, and regulatory considerations remain unresolved. These translational limitations should be carefully considered when interpreting current evidence and designing future microbiome-guided precision oncology studies (Liu et al., 2024).

Translation of microbiome research into clinical oncology is hindered by several unresolved methodological and regulatory barriers. Standardized protocols for stool collection, storage, DNA extraction, sequencing, and bioinformatic analysis are still lacking, resulting in substantial inter-study variability and limiting reproducibility across cohorts. Differences in sequencing platforms, reference databases, analytical pipelines, and statistical methods can generate inconsistent microbial signatures even when similar patient populations are studied. In addition, population heterogeneity related to diet, geography, ethnicity, lifestyle, antibiotic exposure, proton pump inhibitor use, and other medications complicates the identification of universally applicable biomarkers. Regulatory considerations remain particularly challenging for microbiome-based therapeutics, including donor screening requirements for fecal microbiota transplantation, biosafety assessment of engineered microbes, genetic containment strategies, manufacturing under Good Manufacturing Practice standards, and long-term monitoring for ecological and immunological effects. Another major limitation is the limited reproducibility of many published microbiome signatures in external validation cohorts, raising concerns regarding overfitting and restricted generalizability. Consequently, large prospective multicenter studies with harmonized methodologies and predefined clinical endpoints are required before microbiome-guided diagnostics and therapeutics can be reliably incorporated into routine precision oncology practice.

Future studies should depart from taxonomic descriptions and incorporate advanced methodologies focused on functional aspects of host–microbiota interactions. The holistic use of metagenomics, meta transcriptomics, and metabolomics, combined with single-cell methodologies, will elucidate the multifactorial role of microbes and their impact on tumor evolution. Additionally, the nascent domains of synthetic biology, microbiome engineering, and systems medicine may provide the scaffolding for advanced, high-precision therapeutic avenues based on the unique biological realities of each patient (Yang et al., 2025). The combination of multi-omics technologies and artificial intelligence will promote a rapid expansion of new biomarkers and improve the precision of therapies and the molecular stratification of patients (He et al., 2026). The convergence of microbiologists, oncologists, bioinformaticians, and clinical researchers are needed to actualize these concepts. Taking a combined approach and breaking from past habits will be required to understand the barriers of microbiome-guided precision oncology. New developments in this area will most profit from this combination and help patients with new, better, and more effective therapies (Hsu et al., 2025).

## 11 Conclusion

Microbiota research has introduced an additional dimension to tumor biology. It now offers an additional target to include symptomatic responses to tumor aggressiveness and for potential customized treatment options. Microbiota can shift immune responses to a more aggressive form for tumor growth and persistence. The gut microbiota is also able to shift immune responses and tumor growth through the utilization of different microbial metabolites. Tumor persistence is aided further by gut microbiota promoting dysbiosis. Dysbiosis means the change of regular gut microbiota. This shift of gut microbiota can promote cell survival and growth as well as Warburg effect dysregulation and immune response dysregulation. Among recent developments in microbiota and tumor crosstalk is an understanding of microbial membranes vesicles and bacterial RNAs along with ferroptosis and mitochondrial function pathways. These all offer therapeutic targets that are beginning to emerge in a crosstalk manner. Microbiota and tumor crosstalk are able to create a localized model of precision therapeutics to create therapeutic targets.

Microbiota can be utilized to promote tumor treatments and overcome treatment resistance in a personalized manner. The interface of AI with microbiota and tumor treatment research promises the localization of highly precise therapeutics to meet personalized therapeutic targets. Microbiome research is addressing existing issues related to standardization, causality evaluation, and translation to the clinic, and is changing the field of cancer research. The current focus on characterizing functional microbiomes and developing personalized interventions will facilitate the transition from association to clinical application via mechanisms. Jointly, the application of gut microbiome research to therapeutic and diagnostic realms will help develop more precise, effective, and personalized cancer treatment strategies.
